# A Unique Mitochondrial Transcription Factor B Protein in *Dictyostelium discoideum*

**DOI:** 10.1371/journal.pone.0070614

**Published:** 2013-07-26

**Authors:** Sam Manna, Phuong Le, Christian Barth

**Affiliations:** 1 Department of Microbiology, La Trobe University, Melbourne, Victoria, Australia; 2 Tokyo Metropolitan University, Department of Biological Science, Tokyo, Japan; University of Dundee, United Kingdom

## Abstract

Unlike their bacteriophage homologs, mitochondrial RNA polymerases require the assistance of transcription factors in order to transcribe mitochondrial DNA efficiently. The transcription factor A family has been shown to be important for transcription of the human mitochondrial DNA, with some of its regulatory activity located in its extended C-terminal tail. The mitochondrial transcription factor B family often has functions not only in transcription, but also in mitochondrial rRNA modification, a hallmark of its α-proteobacterial origin. We have identified and characterised a mitochondrial transcription factor B homolog in the soil dwelling cellular slime mould *Dictyostelium discoideum,* an organism widely established as a model for studying eukaryotic cell biology. Using *in bacterio* functional assays, we demonstrate that the mitochondrial transcription factor B homolog not only functions as a mitochondrial transcription factor, but that it also has a role in rRNA methylation. Additionally, we show that the transcriptional activation properties of the *D. discoideum* protein are located in its extended C-terminal tail, a feature not seen before in the mitochondrial transcription factor B family, but reminiscent of the human mitochondrial transcription factor A. This report contributes to our current understanding of the complexities of mitochondrial transcription, and its evolution in eukaryotes.

## Introduction

Mitochondria are vital to the eukaryotic cell as they generate the bulk of the energy required for cellular processes. The double membrane organelles have many characteristics in common with bacteria as a result of their evolution from a primitive α-proteobacterium. Interestingly, mitochondria have maintained their own genome, which can vary in size significantly. Most of the genes in the mitochondrial genome encode proteins, which play a role either in oxidative phosphorylation, or are components of the mitochondrial translation machinery [1], [2]. Thus, mitochondria are now heavily reliant on the nucleus of the cell to provide many proteins that are required for mitochondrial function, but not encoded in the mitochondrial genome. These include proteins involved in the maintenance, replication and transcription of the mitochondrial genome.

In most eukaryotes, mitochondrial DNA (mtDNA) is transcribed by a T-odd numbered bacteriophage-like RNA polymerase [3]–[5]. However, in contrast to their bacteriophage counterparts, mitochondrial RNA polymerases (mtRNAPs) require additional proteins to facilitate efficient transcription [2], [5]. This process involves another group of nuclear encoded mitochondrial proteins, the mitochondrial transcription factors. There are two families that, together with the mtRNAP, form the core mitochondrial transcription machinery. The mitochondrial transcription factor A (mtTFA) is a high mobility group containing protein that can bind and bend DNA, making promoter regions more accessible to the mtRNAP [6], [7]. The mtTFA protein has also been found to play roles in the maintenance, copy number and nucleoid formation of mtDNA [8]–[11].

Members of the mitochondrial transcription factor B (mtTFB) family have also been found to be important positive regulators of mitochondrial transcription. Originally believed to be homologous to bacterial sigma factors, most mtTFB proteins are now known to contain an rRNA adenosine dimethyltransferase domain, a hallmark of their α-proteobacterial origin [4], [12], [13], [14]. Some members of the mtTFB family containing this domain have been shown to be responsible for adenosine dimethylation that occurs in a highly conserved stem loop of the mitochondrial small subunit rRNA (*rns*). In metazoans, the *mtTFB* gene has undergone a duplication event, with an evolutionary trend towards separating the two functions into individual proteins. This has been demonstrated with the human mtTFB homologs, h-mtTFB1 and h-mtTFB2 (also known as TFB1M and TFB2M, respectively), which display both functions, but have varied degrees of methyltransferase activity and opposing efficiencies as transcriptional activators [13]–[15]. In addition to its main role as a transcription factor, h-mtTFB2 has also been shown to function in transcription-primed mtDNA replication [16]. In contrast, h-mtTFB1 functions primarily as an rRNA adenosine dimethyltransferase, but also exhibits some transcription factor activity and is also important for translation [13], [14], [15], [17]. The mtTFB homolog from *Saccharomyces cerevisiae*, mtf1, on the other hand, functions solely as a mitochondrial transcription factor and exhibits no methyltransferase activity [15]. Consistent with this, mtf1 lacks methyltransferase domains and yeast mitochondrial *rns* transcripts are not methylated in this manner [18].

Little is known about how mtTFB proteins contribute to mitochondrial transcription, as they often seem to lack obvious DNA binding and activation domains. In human mitochondria, it is believed that h-mtTFB1/h-mtTFB2 interact directly with the mtRNAP POLRMT, which can then recognise the DNA associated mtTFA, TFAM, allowing mitochondrial transcription to be initiated [19], [20]. TFAM contains a C-terminal extended tail, which is recognised by h-mtTFB1/h-mtTFB2, and which has DNA binding and transcription activation functions, making TFAM vital for transcription in human mitochondria [2], [19], [21]. This is a rather simplified description of how transcription occurs in human mitochondria. While the core mitochondrial transcription machinery seems to be conserved amongst species, the specific roles of the individual components can vary. In yeast for example, the mtTFA homolog, Abf2, has little role in mitochondrial transcription [2]. Similarly to yeast, the mtTFA homolog in the fly *Drosophila melanogaster* also has no function in the transcription of mtDNA [22]. Interestingly, it is the mtTFB2 protein from *D. melanogaster* that is the primary activator of transcription and transcription-primed mtDNA replication, while mtTFB1 plays a role in mitochondrial translation [23]–[25]. Thus, in order to contribute to the understanding of mitochondrial transcription in greater detail and to further investigate how this process is regulated in other organisms, we have characterised a mtTFB homolog in the eukaryotic model organism *Dictyostelium discoideum*.

*D. discoideum* is an amoeba which has been well utilised to study many different aspects of eukaryotic cell biology. This is due to its unique life cycle which provides multiple developmental stages to study differentiation and development, while encompassing many features of the humble microbe that make it rather easy to cultivate and manipulate in a laboratory setting [26]. As a result of this, *D. discoideum,* whose entire genome has been sequenced, has also more recently become an attractive model for studying mitochondrial genetics and disease [27]. Mitochondrial transcription in this protist has been found to be initiated from a single site, a unique characteristic given the size of the genome (∼56 kb) [28], [29]. This initiation site is located in a non-coding region (NCR), ∼2 kb in size, which houses a presently unidentified promoter. The mtRNAP in *D. discoideum,* rpmA, is the only *trans* component of the mitochondrial transcription machinery to have been characterised thus far. This phage-like RNA polymerase was found to specifically bind and initiate transcription in the NCR of the *D. discoideum* mitochondrial genome, but does so rather inefficiently [29], suggesting a requirement for mitochondrial transcription factors.

This study characterises a mtTFB homolog in *D. discoideum* which not only functions as a rRNA adenosine dimethyltransferase, but also upregulates mitochondrial transcription in association with rpmA. Additional features of this transcription factor contribute to its transcriptional activation properties, which seem to be lacking in other mtTFB homologs characterised to date, providing new insights into the process of mitochondrial transcription initiation.

## Results

### Identification of a mtTFB Homolog in *D. discoideum*

**Figure 1:**
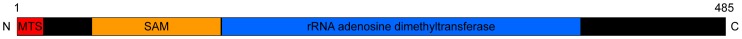
Domain architecture of Tfb1m. Bioinformatic analysis revealed that the 56 kDa protein has an N-terminal mitochondrial targeting signal (MTS), an S-adenosyl-methionine (SAM) binding site, and a rRNA adenosine dimethyltransferase domain, features characteristic of most mtTFB homologs.

**Figure 2:**
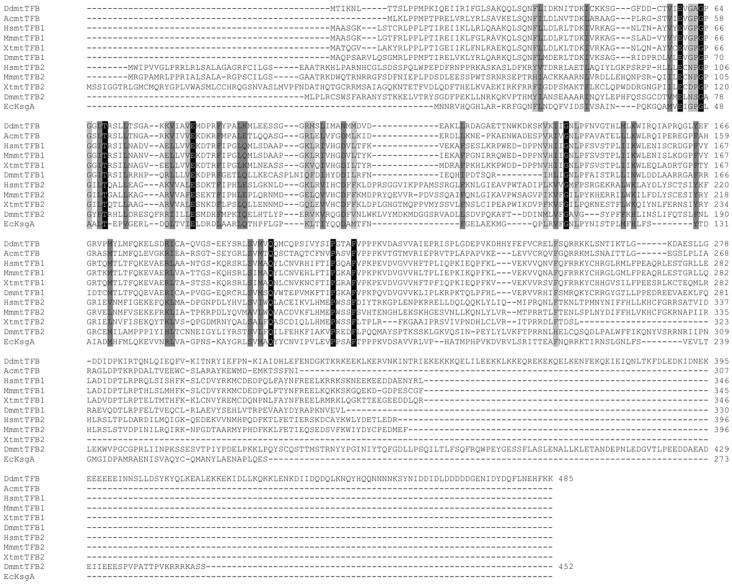
Amino acid sequence alignment of Tfb1m from *Dictyostelium discoideum* (DdmtTFB) with mtTFB homologs. Included are mtTFB homologs from *Acanthamoeba castellanii* (Ac, accession no. ABB97063), *Homo sapiens* (Hs, accession no. NP_057104 and NP_071761), *Mus musculus* (Mm, accession no. NP_666186 and NP_032275), *Xenopus (Silurana) tropicalis* (Xt, accession no. NP_001016494 and AAI27364), *Drosophila melanogaster* (Dm, accession no. Q9VTM5 and Q9VH38) and KsgA from *Escherichia coli* (Ec, accession no. YP_488357). Amino acids shaded in black indicate identical amino acids, while conserved and semi-conserved amino acids are depicted in dark and light grey, respectively.

A putative *mtTFB* gene sequence in *D. discoideum* had been identified by Shutt and Gray, 2006 [4]. However, the sequence was only used for phylogenetic analyses and was not characterised further. Recently we have analysed the *D. discoideum* genome in a search for any mitochondrial transcription factor gene sequences leading to the identification of only one candidate (Genbank accession no. XM_632683), which was the same as the one identified previously. This gene, which we have named *tfb1m,* is 1458 bp in size and consists of a continuous open reading frame. The corresponding protein sequence is 485 amino acids long, has a predicted molecular weight of ∼56 kDa, and contains a putative N-terminal mitochondrial targeting signal (Fig. 1). It also has a predicted S-adenosyl-methionine (SAM) binding site and a rRNA adenosine dimethyltransferase domain and the amino acid sequence displays homology to mtTFB proteins in other organisms, as well as to rRNA adenosine dimethyltransferases in bacteria (Fig. 2).

### Tfb1m Localises to Mitochondria

**Figure 3:**
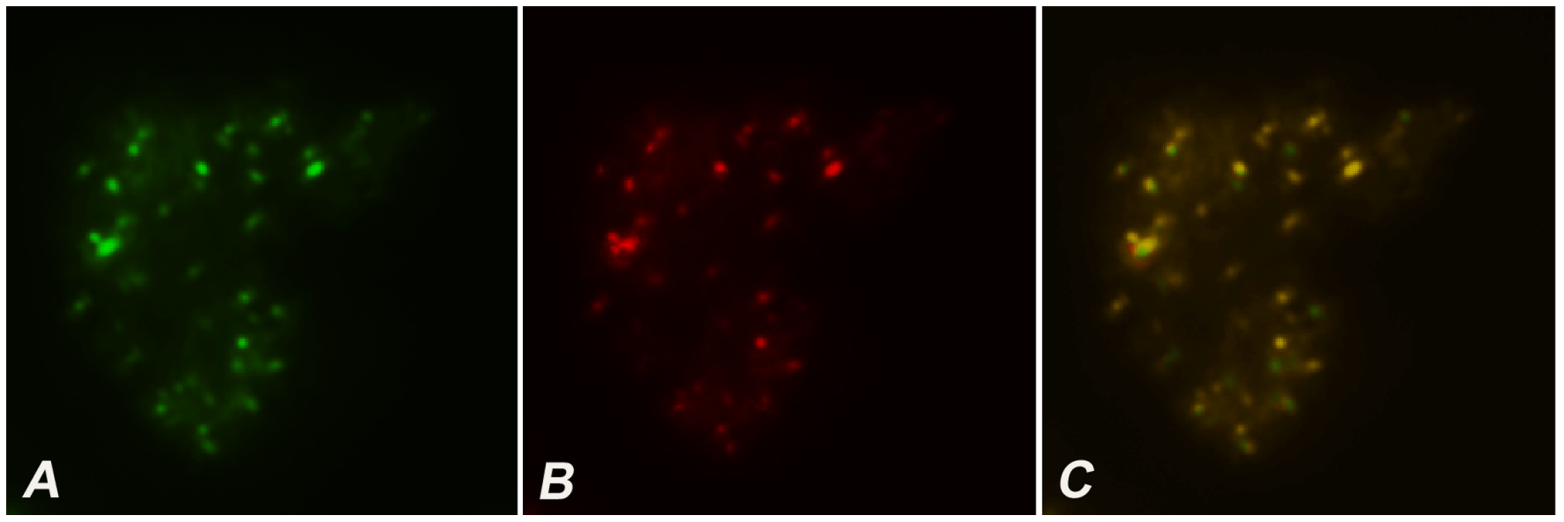
Mitochondrial localisation of Tfb1m. Fluorescence microscopy of (A) *D. discoideum* cells expressing a Tfb1m:GFP fusion protein and (B) stained with Mitotracker, indicating that the fusion protein and the mitochondria co-localise (C).

Mitochondrial targeting of Tfb1m was suggested by high probability scores obtained using the software programs Mitoprot (probability score: 0.71) [30] and Mitopred (probability score: 0.85) [31]. To confirm the localisation of Tfb1m, a fusion gene was created consisting of the 5′ end of *tfb1m* (846 bp), encoding the putative mitochondrial targeting signal, fused to the gene encoding the green fluorescent protein (GFP) in the *D. discoideum* shuttle vector pA15GFP [32]. When this construct was introduced into *D. discoideum* cells, the encoded fusion protein was found to co-localise with mitochondrial markers (Fig. 3), indicating that the N-terminal portion of Tfb1m was able to target the fusion protein to the mitochondria, demonstrating that Tfb1m is in fact a mitochondrial protein.

### Tfb1m Displays rRNA Adenosine Dimethyltransferase Activity in *Escherichia Coli*

**Figure 4:**
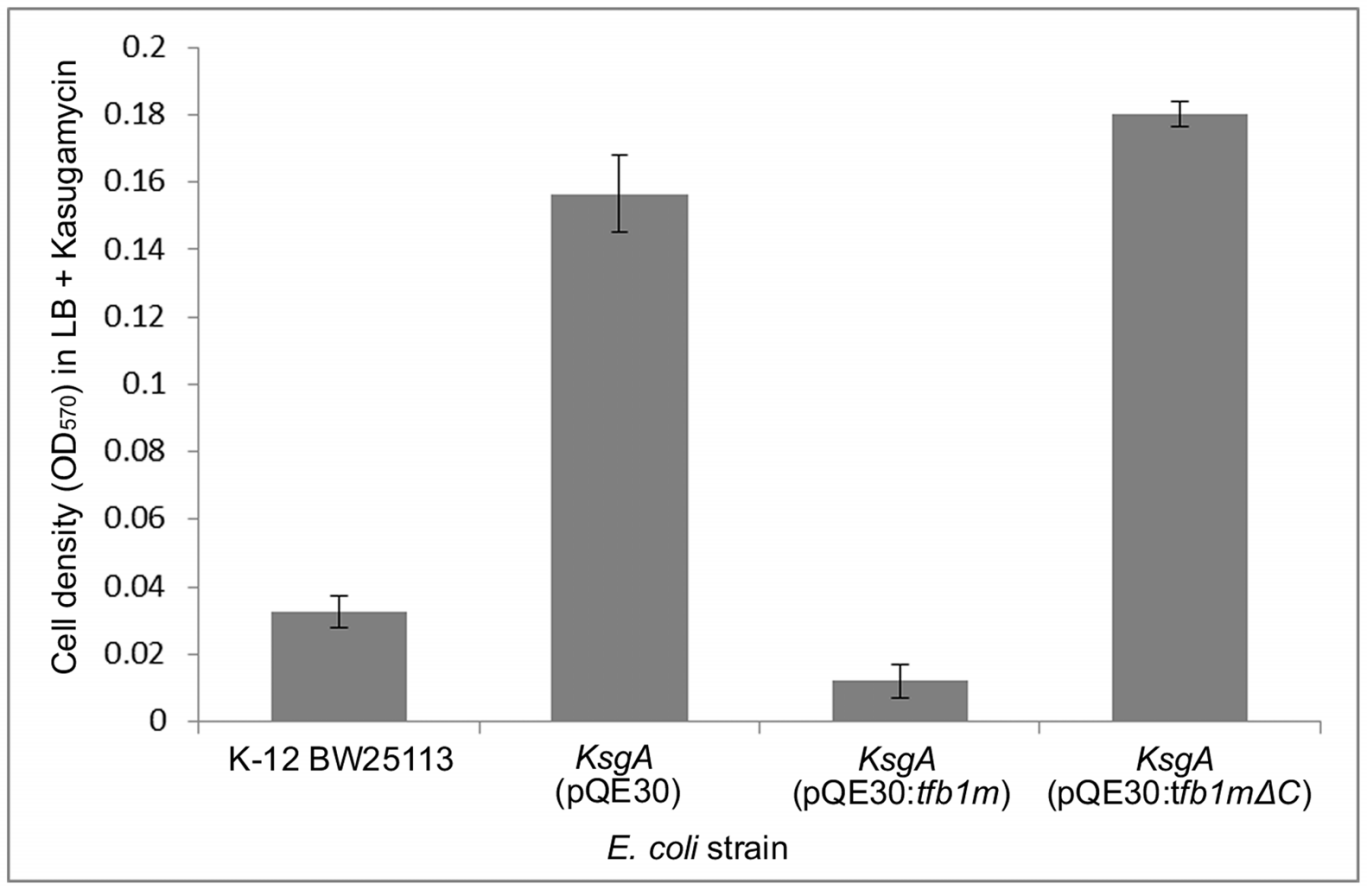
Analysis of kasugamycin resistance to evaluate rRNA adenosine methyltransferase activity. Cell densities of an *E. coli ksgA* mutant (*KsgA*[pQE30], second column), compared to its parental strain K-12 BW25113 (first column) and the same mutant expressing either the *D. discoideum* mitochondrial transcription factor Tfb1m (*KsgA*[pQE30:*tfb1m*], third column), or a C-terminally truncated variant of Tfb1m (*KsgA*[pQE30:*tfb1m*Δ*C*], fourth column). Cells were grown in LB supplemented with ampicillin (100 µg/mL) and kasugamycin (800 µg/mL) at 37°C for 15 h, and growth was analysed by measuring the optical density of the cultures.

The mtTFB family is hypothesised to have evolved from an α-proteobacterial rRNA adenosine dimethyltransferase belonging to the KsgA family [33]. Given that it has been shown that h-mtTFB1 and h-mtTFB2 have retained rRNA methyltransferase activity in human mitochondria [15], [34], we investigated if Tfb1m also possessed this activity. Studies into KsgA in *Escherichia coli* have demonstrated that the knockout of its gene results in a lack of methylated 16S rRNA and, as a consequence of this, resistance to kasugamycin, an aminoglycoside that functions by interacting with 16S rRNA, the substrate for KsgA [35]–[37]. Multiple studies have utilised this property to study rRNA methyltransferase activity of mtTFB proteins in *E. coli*, where introduction of a mtTFB encoding gene into a *ksgA* mutant restored methylation of 16S rRNA and therefore sensitivity to kasugamycin [15], [34], [38]. The restoration of this sensitive phenotype was also observed when the *D. discoideum* Tfb1m was expressed in *E. coli ksgA* mutants. The ability of Tfb1m-expressing *E. coli ksgA* mutant cells to grow in the presence of kasugamycin was reduced significantly compared to their highly resistant phenotype in the absence of Tfb1m (Fig. 4). The restored sensitivity to kasugamycin suggests that Tfb1m is capable of complementing KsgA *in bacterio* in the methylation of 16S rRNA, and hence must have a rRNA methyltransferase activity similar to KsgA. Conversely, *ksgA* mutants expressing a C-terminally truncated form (193 amino acids) of Tfb1m (Tfb1mΔC) remained resistant to kasugamycin, despite the fact the methyltransferase domain remained intact. Thus, although the C-terminus does not carry any enzymatic activity, its deletion abolishes the methyltransferase activity of Tfb1m, suggesting that the C-terminus is still required for methylation.

### The Presence of Tfb1m Activates Mitochondrial Transcription *in Bacterio*

**Figure 5:**
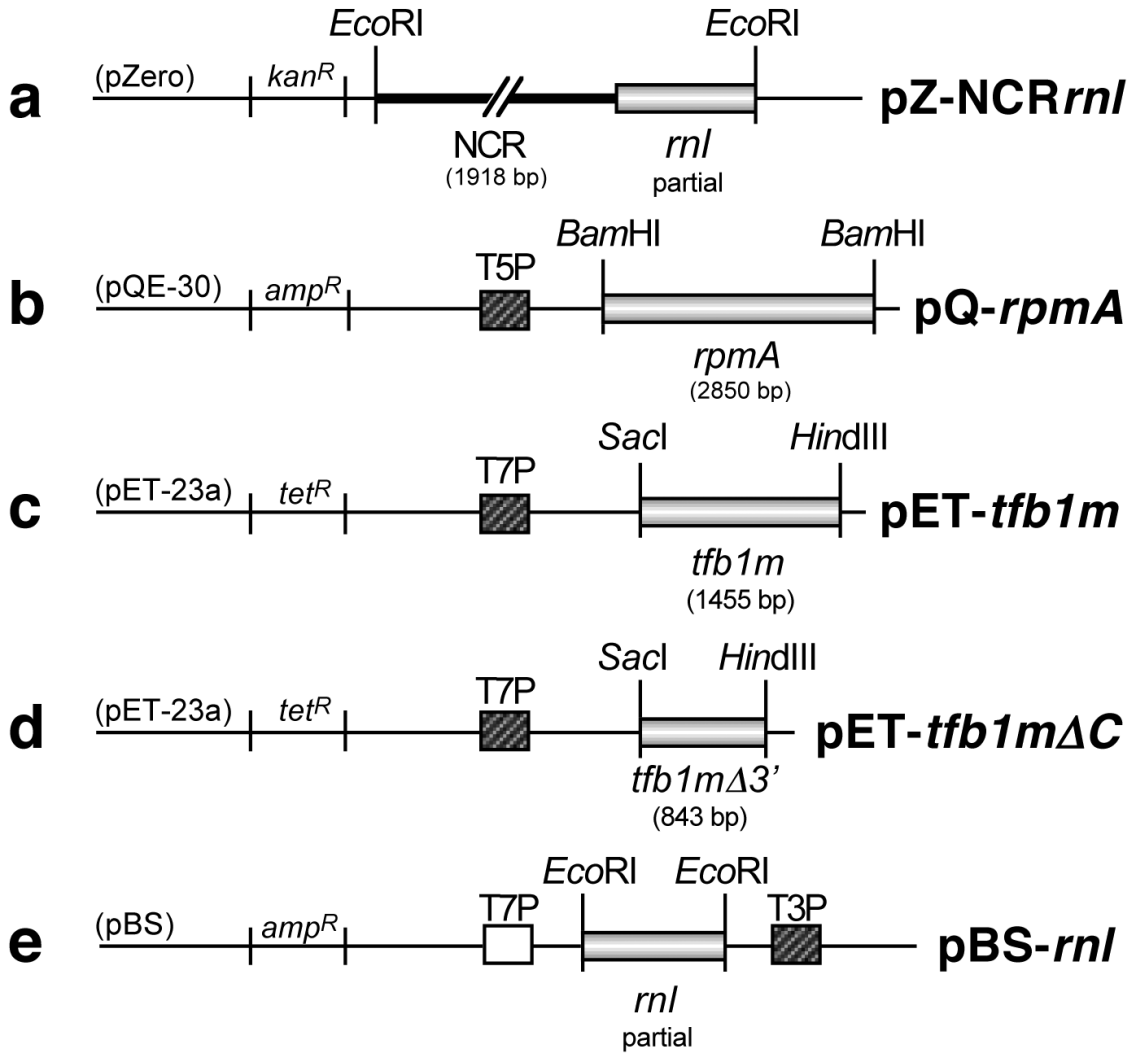
Schematic representation of vector constructs used for *in bacterio* transcription experiments. a) Vector pZ-NCR*rnl* contains the non-coding region (NCR) including the transcription initiation site of the *D. discoideum* mitochondrial genome, and the partial sequence of the *rnl* gene downstream of the initiation site, which serves as a transcriptional reporter. The vector confers resistance to kanamycin. b) Vector pQ-*rpmA* contains the *rpmA* gene under the control of a T5*_lac_* promoter/operator for heterologous protein expression. The vector confers resistance to ampicillin. c) Vector pET-*tfb1m* contains the full length *tfb1m* gene under the control of a T7 promoter. The vector confers resistance to tetracycline. d) Vector pET-*tfb1mΔC* contains the *tfb1m* gene lacking the 3′ end of the gene encoding the C-terminus. The vector confers resistance to tetracycline. e) Vector pBS-*rnl* contains the partial sequence of the mitochondrial *rnl* gene, which was used for *in vitro* transcription to generate a radiolabelled antisense *rnl* RNA probe transcribed from the T3 promoter. Constructs pZ-NCR*rnl,* pQ-*rpmA* and pBS-*rnl* were obtained from Le et al., 2009 [29].

**Figure 6:**
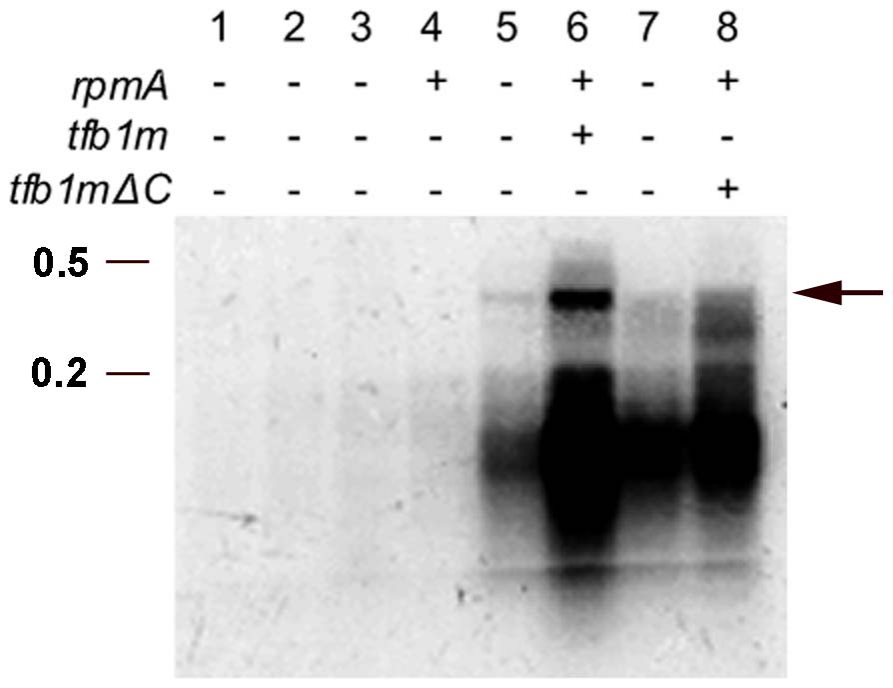
Analysis of *in bacterio* transcribed RNA. Northern hybridisation using an antisense *rnl* probe against total RNA isolated from *E. coli* cells harbouring vector pZ-NCR*rnl* (lanes 1 and 2), vectors pZ-NCR*rnl* and pQ-*rpmA* (lanes 3 and 4), vectors pZ-NCR*rnl,* pQ-*rpmA* and pET-*tfb1m* (lanes 5 and 6), or vectors pZ-NCR*rnl,* pQ-*rpmA* and pET-*tfb1mΔC* (lanes 7 and 8). Heterologous expression of the *D. discoideum* mitochondrial RNA polymerase (rpmA) from pQ-*rpmA* and of the mitochondrial transcription factor Tfb1m from pET-*tfb1m* or pET-*tfb1mΔC* is indicated by+(induced) or – (non-induced). The top bands (∼ 420 nucleotides long) indicated by the arrow represent genuine transcription products initiated at the mitochondrial transcription start site. The smear below may be the result of partially degraded or prematurely terminated transcription products. RNA size markers are indicated in kilo bases to the left of the panel.

To determine if Tfb1m plays a role in mitochondrial transcription, we assessed the function of this protein in a previously established *in bacterio* transcription system for analysing mitochondrial transcription. This system has been developed as an alternative to studying *D. discoideum* mitochondrial transcription *in vitro*, which proved to be problematic for several reasons as described previously [39], [40]. The *in bacterio* transcription system involves the use of an *E. coli* BL21 DE3 strain harbouring two vectors (Fig. 5). The first, pZ-NCR*rnl*, contains the NCR including the transcription initiation site of the *D. discoideum* mitochondrial genome, as well as the partial sequence of the mitochondrial *rnl* gene, which serves as a transcriptional reporter (Fig. 5A). The second vector, pQ-*rpmA,* encodes the *D. discoideum* mtRNAP (rpmA, Fig. 5B) and allows inducible expression of the protein in the presence of IPTG. Hence, when expression of rpmA is induced in the *E. coli* host, the polymerase will recognise its binding site in the NCR and initiate transcription of the *rnl* gene from pZ-NCR*rnl* [29]. We modified the existing system by introducing a third vector into these cells, encoding Tfb1m. This vector, pET-*tfb1m* (Fig. 5C) was constructed using the expression vector pET-23a*TetA*, a derivative of the pET-23a(+) vector (Novagen), in which the selectable marker has been changed to make it suitable for this system [41]. For the transcription experiment, rpmA was expressed (from vector pQ-*rpmA*) in the presence or absence of Tfb1m (expressed from vector pET-*tfb1m*) in *E. coli* cells harbouring pZ-NCR*rnl*. The bacterial RNA was extracted, separated on agarose gels, and blotted onto a nylon membrane to detect any *rnl* transcript using an *rnl* specific probe. *E. coli* cells carrying only pZ-NCR*rnl* acted as a negative control and demonstrated that any transcription products derived from this construct were not due to endogenous RNA polymerase activity. Bacterial cells harbouring only pZ-NCR*rnl* and pQ-*rpmA* did not express any RNA that hybridised to the *rnl* specific probe. Transcription of the reporter gene from pZ-NCR*rnl* was only detected in cells expressing both rpmA and Tfb1m (Fig. 6). This clearly showed that the *D. discoideum* transcription factor functions as a positive regulator of mitochondrial transcription in *D. discoideum.*

### A Unique Extended C-terminal Tail in Tfb1m Contributes to its Role in Transcription

**Figure 7:**
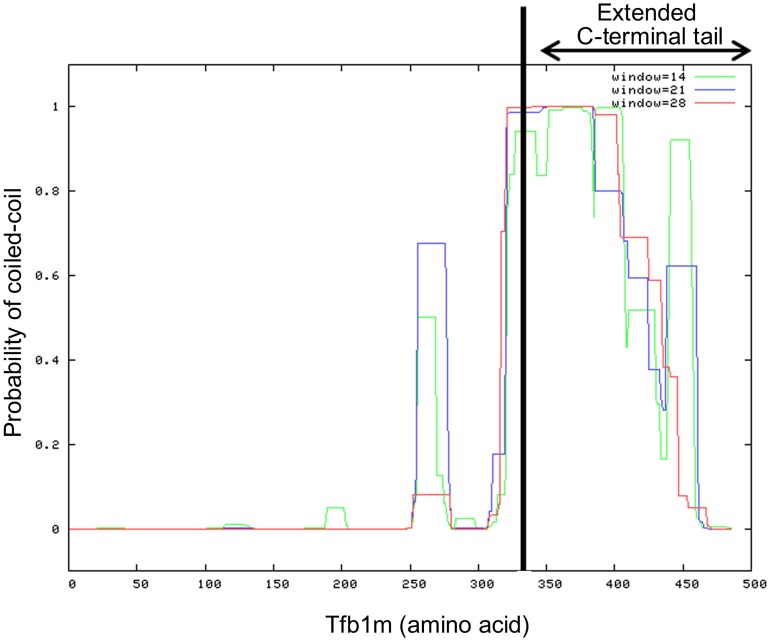
Coiled-coil motif prediction analysis for Tfb1m. Analysis was performed using the bioinformatic program Coils [42] at 14 (green), 21 (blue) and 28 (red) amino acid frames.

*In silico* analysis of Tfb1m revealed that the protein is larger than its homologs in other species, and that it contains an extended C-terminal tail not present in other mtTFB proteins. Compared to most mtTFB proteins in other organisms, the C-terminus of Tfb1m extends by an additional 65–145 amino acids (Fig. 2). Secondary structure analysis of Tfb1m using the bioinformatic program Coils [42] indicated that part of this tail can form a coiled-coil structure (Fig. 7). A high probability for coiled-coil formation in the C-terminus was also supported by Paircoil [43], a second coiled-coil predictive program (data not shown). To identify the functional significance of this extended C-terminal tail, a vector was created with a 579 bp deletion from the 3′ end of the *tfb1m* gene encoding a truncated form of Tfb1m. The resulting construct, pET-*tfb1mΔC* (Fig. 5D), was then used in the above described *in bacterio* transcription system to replace the pET-*tfb1m* construct encoding the full length protein. The absence of the C-terminal tail was found to reduce the transcription of the reporter gene significantly as compared to when the full length Tfb1m was present (Fig. 6). This demonstrated that in addition to methylation, the extended C-terminal tail of Tfb1m must also have an important role in transcriptional activation.

## Discussion

### Tfb1m, a Mitochondrial Protein with Dual Function

We have characterised a mtTFB homolog in the cellular slime mould *D. discoideum* with two distinct functions. First, the kasugamycin resistance assay revealed that Tfb1m can functionally replace KsgA in *E. coli* (Fig. 4), indicating that Tfb1m must have a methyltransferase activity similar to KsgA. KsgA forms part of the bacterial family of adenosine dimethyltransferases responsible for the dimethylation of two adenosine residues located in a highly conserved stem loop at the 3′ of the small subunit rRNA. Based on the functional similarities between the bacterial and the *D. discoideum* protein, and the fact that the *D. discoideum* protein can supplement KsgA function in *E. coli,* Tfb1m is likely to be involved in the dimethylation of adenosine residues in the highly conserved stem loop region at the 3′ end of the small subunit rRNA in *Dictyostelium* mitochondria.

The second function of Tfb1m was demonstrated using an *in bacterio* transcription assay, where the protein was found to play a significant role in mitochondrial transcription initiation. Its presence allowed the heterologously expressed mitochondrial RNA polymerase (rpmA) to efficiently initiate transcription at a mitochondrial-specific transcription initiation site, resulting in the expression of a reporter gene (Fig. 6). In previous experiments, rpmA alone was shown to be sufficient for mitochondrial transcription at very low levels [29], making it difficult to detect any transcription activity via northern blot hybridisation. In the current study, we could not detect any transcription of the reporter gene with only rpmA expressed in the *E. coli* cells. Only when the polymerase was expressed together with Tfb1m in the same cells, did the expression of the reporter gene rise to detectable levels. This clearly suggests the need for transcription factors by rpmA, a characteristic feature of most mtRNAPs [2], [5]. The fact that we did not observe any transcription activity at all with rpmA alone in our experiments may have been due to difficulties in detecting small amounts of transcripts, but made the difference in transcription levels in the absence or presence of Tfb1m even more obvious. The sharp differences in transcription efficiencies in the absence or presence of Tfb1m demonstrated the dependence of the RNA polymerase on this transcription factor, and also suggest that Tfb1m may be the primary and possibly the sole mitochondrial transcription factor in *D. discoideum.*

Our results clearly demonstrated that Tfb1m is a dual function protein with roles in *rns* methylation and in the positive regulation of mitochondrial transcription. This is in contrast to the *S. cerevisiae* homolog, mtf1, which has been shown to function solely as a transcription factor [15]. On the other hand, the dual function of Tfb1m is consistent with the function of the human homologs, h-mtTFB1 and h-mtTFB2, which both display varied levels of methyltransferase activity and transcriptional activation [15]. Given that *rns* methylation in human mitochondria by h-mtTFB1 has been shown to consequently affect ribosome biogenesis and translation efficiency via interactions with the human mitochondrial RNA polymerase [16], [44], it is tempting to postulate that the *rns* methylation by Tfb1m in *D. discoideum* mitochondria may serve similar functions. In addition to the postulated role in translation, the mtTFB homologs in humans and in *D. melanogaster* have been suggested to have a role in mitochondrial DNA replication [16], [23]. Whether Tfb1m also contributes to these processes in *D. discoideum* mitochondria remains to be elucidated.

### The Significance of an Extended C-terminal Tail in Tfb1m

Detailed analyses of the Tfb1m sequences identified an extended C-terminal tail (∼145 amino acids), which, as our results demonstrated, contributes to the activator function of the transcription factor (Fig. 6). Approximately half of this extended tail displays the ability to form a coiled-coil structure (Fig. 7), a protein folding motif that has been identified in many different proteins with diverse functions in processes such as cell organisation and movement, signal transduction pathways, and vesicle trafficking [45], [46]. However, coiled-coil structures have also been identified in a number of nuclear transcription factors, such as the basic region-leucine zipper proteins, and, based on this, coiled-coil domains have been implicated in several transcriptional related functions [45], [46]. It is therefore likely that the C-terminal coiled-coil domain in Tfb1m possesses similar functional properties, which was supported by the reduced transcriptional activity of rpmA when the C-terminal coiled-coil region of Tfb1m had been deleted (Fig. 6). The lack of a distinct transcript and the increased transcript degradation or premature termination in *E. coli* cultures expressing the C-terminal deletion variant of Tfb1m also implies that in addition to its transcription activation properties, the extended C-terminal tail may also function in stabilising nascent transcripts or may regulate termination of transcription. Additionally, given that deletion of the C-terminus also abolished methylation in *E. coli* (Fig. 4), it seems that the functions of Tfb1m in methylation and in transcription may be linked, possibly through the coiled-coils, which partially overlap with the methyltransferase domain (Fig. 7). The coiled-coils may be required for correct folding of the methyltransferase domain, or may be directly involved in the activation of the transcription factor.

To our knowledge, this is the first report of a mtTFB homolog containing an extended and coiled-coil forming C-terminal tail with a role in transcription. While it is known that the C-terminus of the yeast mtTFB homolog also has a function in mitochondrial transcription, the overall length of the protein (341 amino acids compared to 485 amino acids for Tfb1m) is comparable to that of other mtTFB homologs and the C-terminus is not extended [47]. Additionally, no coiled-coil regions have been reported in the C-terminus of the yeast homolog and we were also unable to identify any such structures (data not shown). Our sequence comparisons showed that the mtTFB2 homolog from *D. melanogaster* also possesses a shorter, but nonetheless extended C-terminal tail (∼80 and ∼145 amino acids in *D. melanogaster* mtTFB2 and *D. discoideum* Tfb1m, respectively, as compared to other mtTFB proteins) (Fig. 2). Although the C-terminus in the *D. melanogaster* protein also has the potential to form coiled-coil structures (data not shown), the probability scores for coiled-coil formation were not as significant as for Tfb1m. In addition, the significance of the C-terminal tail in the *D. melanogaster* homolog in regulating mitochondrial transcription remains to be elucidated. The excessive length of the C-terminal tail and the presence of coiled-coil structures in Tfb1m clearly distinguishes it from other mtTFB homologs, suggesting that *D. discoideum* uses a unique mechanism for regulating mitochondrial transcription.

**Figure 8:**
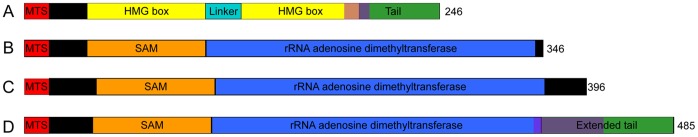
Comparison of the *D. discoideum* mitochondrial transcription factor to the human mitochondrial transcription factors. Displayed are the domain architectures of human mitochondrial transcription factors TFAM (A), h-mtTFB1 (B) and h-mtTFB2 (C) and the *D. discoideum* mitochondrial transcription factor Tfb1m (D). Purple overlapping regions denote coiled-coil regions.

While C-terminal extensions do not seem to be common in the mtTFB family, the human mitochondrial transcription factor TFAM, a member of the mtTFA family, possesses such an extended C-terminal tail [2], [19], [21]. This tail, although significantly shorter (∼30 amino acids) as compared to that in Tfb1m, is also predicted to form coiled-coil structures [9], [10]. Most of the transcriptional activator properties of TFAM have been attributed to this tail including DNA binding, homodimerisation and recruitment of the h-mtTFB2/POLRMT complex to the transcription start site via specific interactions of h-mtTFB1 or h-mtTFB2 with the C-terminal tail of TFAM [19], [20]. This not only supports the contribution of the C-terminal tail of Tfb1m to its function in transcription activation, but also implies that it may do so by interacting with other proteins of the transcription machinery. Additionally, our search for a mtTFA homolog in *D. discoideum* yielded no candidates (data not shown), indicating that *D. discoideum* may not have a mtTFA homolog. Thus, the lack of a mtTFA homolog, and the presence of an extended coiled-coil forming C-terminal tail in Tfb1m, may mean that *D. discoideum* has developed an alternative mechanism for mitochondrial transcription involving only two proteins, an RNA polymerase and a single transcription factor that combines the features of both mitochondrial transcription factor families (Fig. 8).

### Concluding Remarks

By identifying and characterising the functions of Tfb1m in *D. discoideum,* we have learned more about mitochondrial transcription and its regulation in Eukarya. Although the mitochondrial transcription machinery seems to be conserved to some extent, our studies further demonstrate that differences do exist between species. These include the differences seen in the structures and features of proteins belonging to the same group of transcription factors as well as differences in the types and number of proteins involved. Whether Tfb1m and its function in transcription is a characteristic feature of protozoan mitochondrial transcription in general, or simply unique to *D. discoideum* still remains to be determined.

## Materials and Methods

### Strains and Culture Conditions

*D. discoideum* AX2 strain, a derivative of the NC4 parental strain, and all subsequent transformants, were grown in HL-5 axenic medium [48], [49] at 21°C to a density of 2–5×10^6^ cells/mL. Alternatively, AX2 and all transformant derivatives were grown on SM plates with *Klebsiella aerogenes* lawns for non-axenic cultivation [50].

### Transformation of *D. discoideum* with Vector DNA

Transformation of *D. discoideum* with vector DNA was performed using the calcium phosphate precipitation method as described previously [51] using 20 µg of vector DNA. Transformants were isolated from *Micrococcus luteus* PRF3 lawns on SM plates supplemented with 20 µg/mL G-418, to select for cells that had taken up the vector [52]. Following isolation, transformants were maintained either axenically in HL-5 medium or on *K. aerogenes* lawns on SM agar plates.

### Fluorescence Microscopy

To determine the subcellular localisation of Tfb1m, *D. discoideum* cells transformed with a Tfb1m:GFP fusion vector were analysed via fluorescence microscopy based on protocols described previously [53], [54]. Aliquots of the axenically grown transformant culture (∼3 mL) were transferred into a 6 well plate (BD Biosciences) containing coverslips and the cells were allowed to settle. The medium was removed and the mitochondria were stained with 100 nM Mitotracker (Life Technologies) in Lo-Flo HL-5 medium for 1 h. Unbound Mitotracker was removed by washing the cells with Lo-Flo HL-5 four times, and the cells were subsequently washed twice with phosphate buffer. Cells were fixed by placing coverslips onto a 1% agarose gel in phosphate buffer containing 3.7% paraformaldehyde for 15 min and subsequently washed with phosphate buffered saline four times. Coverslips were rinsed with milli-Q sdH_2_O and mounted for microscopy with 90% glycerol in PBS.

### Kasugamycin Resistance assay

Kasugamycin resistance was assayed to ascertain the methyltransferase activity of Tfb1m using a protocol adapted from Seidel-Rogol et al., 2003 [34] and from Cotney and Shadel, 2006 [15]. An *E. coli ksgA* mutant and its parental strain created by Baba et al., 2006 [55] and provided by the Coli Genetic Stock Centre were used. Overnight cultures were diluted 100 fold in LB broth supplemented with 100 µg/mL ampicillin and incubated at 37°C to an OD_600_ of 0.2. The exponentially growing cultures were diluted to an OD_600_ of 0.01 in LB supplemented with ampicillin (100 µg/mL) and kasugamycin (800 µg/mL). The diluted cultures were then aliquoted into a 96 well microtiter plate (BD Biosciences) in triplicate and incubated at 37°C for 15 h. Growth was analysed by measuring the OD_570_ on a Lucy 2 microtiter plate reader (Quantum Scientific).

### Analysis of Mitochondrial Transcription *in Bacterio*

Analysis of mitochondrial transcription was carried out using the method modified from Le et al., 2009 [29] and Accari et al., 2013 [40]. Following incubation of *E. coli* cultures of interest, 0.02 vol of overnight cultures were inoculated into LB supplemented with the relevant combination of antibiotics (100 µg/mL ampicillin, 25 µg/mL kanamycin, 5 µg/mL tetracycline) to an OD_600_ of 0.4–0.6. Expression of heterologous proteins was then induced via the addition of 1 mM IPTG and cultures were incubated at 21°C for 5 h. In parallel, another set of the same cultures were set up in the same manner, but without the addition of IPTG to serve as un-induced controls. Following *in bacterio* transcription, cultures were pelleted in preparation for RNA extraction. Transcription of the *rnl* reporter was detected via northern blot hybridisation using an *in vitro* transcribed radiolabelled RNA probe for the mitochondrial *rnl*.

### RNA Extraction

Extraction of total RNA from bacterial cultures was performed using TRIzol® reagent (Life Technologies). Cell pellets were treated with 200 µg/mL of lysozyme (Sigma) for 30 min to degrade the cell wall, enhancing cell lysis and RNA yield. Following lysozyme treatment, samples were resuspended in TRIzol® reagent, 0.2 vol of chloroform was added and the samples were mixed vigorously. Samples were then centrifuged to separate the RNA from DNA and proteins. The aqueous phase was collected, and the RNA was precipitated in an equal vol of isopropanol, and centrifuged as previously. RNA pellets were washed with 75% ethanol, air dried and resuspended in DEPC treated sdH_2_O.

### Preparation of Radiolabelled RNA Probes

Synthesis of gene-specific radiolabelled RNA probes was performed via *in vitro* transcription of RNA using a pBS-*rnl* vector to create an antisense *rnl* RNA probe. The reaction consisted of; 5× transcription buffer (Promega): 4 µL, 100 mM DTT: 2 µL, RNasin (Promega) (40 U): 0.5 µL, 2.5 mM rNTPs (rATP, rGTP and rUTP): 3 µL, 100 µM rCTP: 2.4 µL, [α-^32^P] rCTP (10 mCi/mL): 5 µL, linearised pBS-*rnl* vector: 1–2 µg, T3 RNA polymerase (Promega): 1 µL and DEPC sdH_2_O (to 20 µL). The reaction was incubated at 37°C for 1 h and the transcribed RNA was precipitated with an equal volume of isopropanol and used for hybridisation.

### Northern Blot Hybridisation

Northern blot hybridisation was performed similarly to that described previously [56], [57] with the following modifications. Total *E. coli* RNA samples were fractionated on a 2% Tris-acetate (TAE) agarose gel and subsequently transferred to a nylon membrane (Hybond). Following transfer, the membrane was probed with a radiolabelled RNA probe and incubated at 55°C overnight. The blot was exposed to a PhosphorImager screen, and radioactivity was detected using the Amersham Storm 860 Imaging system.
